# Preoperative Oral Tasipimidine in Dogs Undergoing Elective Ovariectomy: Anxiolysis, Handling Quality and Cardiovascular Effects

**DOI:** 10.3390/vetsci13070618

**Published:** 2026-06-26

**Authors:** Nerea Cambeiro-Camarero, Silvia Fernández-Martín, Antonio González-Cantalapiedra

**Affiliations:** 1 Department of Anatomy, Animal Production and Veterinary Clinical Science, Faculty of Veterinary, University of Santiago de Compostela, 27002 Lugo, Spain; antonio.cantalapiedra@usc.es; 2 Rof-Codina Veterinary Teaching Hospital, Faculty of Veterinary, University of Santiago de Compostela, 27002 Lugo, Spain

**Keywords:** tasipimidine, anesthesia, ovariectomy, preoperative anxiety, sedation, recovery, dexmedetomidine, propofol

## Abstract

Preoperative anxiety may compromise perioperative welfare and anesthetic management in dogs. This randomized, blind clinical study evaluated the effects of oral tasipimidine on anxiety-related behaviors, handling quality, cardiovascular variables, anesthetic requirements and recovery in dogs undergoing elective ovariectomy. Thirty ASA I dogs were randomly allocated into three groups (*n* = 10). Treatment groups received tasipimidine (30 µg/kg PO) 45 min before admission, followed by morphine (0.3 mg/kg IM) combined with dexmedetomidine at either 2.5 µg/kg (GTa) or 5 µg/kg IM (GTb). Control dogs (GC) received an oral placebo followed by dexmedetomidine (5 µg/kg IM) and morphine (0.3 mg/kg IM). Anxiety was assessed using the Clinic Dog Stress Scale (CDSS), Visual Analog Scale (VAS), and Reactivity Evaluation Form (REF). Handling quality was assessed using a Numerical Rating Scale (NRS), and recovery quality using the Simple Descriptive Scale (SDS). Tasipimidine significantly reduced anxiety-related behaviors compared with controls, with lower CDSS (5 [0–10] vs. 13 [3–19]), VAS (4 [2–7] vs. 6.5 [2–9]), and REF scores (1 [1–3] vs. 2 [1–4]) (*p* < 0.05). Handling quality improved significantly in treated dogs with lower NRS (1 [1–2] vs. 2 [1–4]; *p* = 0.003). Preoperative heart rate was lower in tasipimidine-treated dogs (85 ± 19 bpm) than in controls (127 ± 23 bpm; *p* < 0.001). Recovery agitation requiring dexmedetomidine rescue sedation occurred in 30% of treated dogs versus 60% of controls. Propofol and fentanyl requirements did not differ significantly among groups (*p* > 0.05). Tasipimidine improved anxiety-related behavioral scores and handling quality in dogs; however, its cardiovascular effects and perioperative role warrant further investigation.

## Simple Summary

Dogs commonly experience fear and stress when admitted to veterinary hospitals for surgical procedures. Increased stress may negatively affect animal welfare and can make handling and preoperative preparation more difficult for veterinary staff. Tasipimidine is an orally administered selective α2-adrenoceptor agonist developed to reduce fear- and anxiety-related behaviors in dogs. This study evaluated whether administering tasipimidine before hospital admission could improve perioperative management in healthy dogs undergoing elective ovariectomy. Dogs receiving tasipimidine showed lower scores on behavioral assessments and were easier to handle during preoperative examination and preparation than dogs receiving placebo. However, these behavioral improvements were not associated with clear differences in sedation, anesthetic drug requirements, intraoperative rescue analgesia, or recovery quality. Lower heart rate values were observed in tasipimidine-treated dogs, although the clinical relevance of these cardiovascular changes remains uncertain because cardiovascular function was not comprehensively assessed. These findings suggest that oral tasipimidine may be useful for improving patient manageability and reducing observable stress-related behaviors before surgery. Further studies are needed to determine whether these behavioral effects provide additional perioperative benefits and to further characterize the cardiovascular effects of the drug.

## 1. Introduction

Fear and anxiety are major stressors that frequently occur during veterinary clinic visits [1,2], affecting approximately 20–50% of dogs, regardless of geographic region [3,4,5]. However, although several pharmacological approaches with gabapentin, dexmedetomidine, and trazodone [6,7,8,9,10,11,12,13] have been investigated in veterinary practice, the incidence, clinical relevance, and standardized management of preoperative anxiety remain poorly characterized. Consequently, anxiety should be considered a key factor to address in both clinical practice and veterinary anesthesia [14].

In human medicine, preoperative anxiety is a well-recognized phenomenon associated with fear of surgery, anesthesia, and awakening [15,16]. Preoperative anxiety activates stress response, increasing glucocorticoid secretion, elevating the risk of postoperative infection, delaying wound healing [17,18], and correlating with higher requirements for anesthetic gases and propofol [19]. It is also associated with increased postoperative morbidity and more challenging pain management [15].

Similarly, in dogs, fear and anxiety induce changes in neurotransmitter and hormone levels that negatively affect the immune, cardiovascular, and metabolic systems [20,21]. Furthermore, handling anxious animals poses additional risks to veterinary staff [22,23].

In humans, preoperative anxiety is managed using both non-pharmacological and pharmacological approaches [17]. Non-pharmacological strategies include music therapy [24,25], cognitive-behavioral therapy [26], and relaxation techniques [16,27], whereas pharmacological treatment involving anxiolytics, such as benzodiazepines and pregabalin [28,29], is typically administered as a single preoperative dose rather than routinely [30].

In veterinary practice, “pet-friendly” handling techniques, including environmental modifications and music therapy [31], combined with anxiolytic medication, have been employed to reduce anxiety in clinical settings [32,33]. However, the effectiveness of non-pharmacological interventions, such as music therapy, remains controversial [34], reflecting similar uncertainty in human medicine [35].

Although calm handling and vocal reassurance may help reduce anxiety in animals, pharmacological strategies remain important tools for managing fear and anxiety, particularly in highly stressed or anxious animals. Among the most used and effective agents are trazodone [36], gabapentin [37], transmucosal dexmedetomidine [38], and alprazolam [37].

Despite their widespread use, evidence regarding the perioperative effects of anxiolytic strategies in veterinary anesthesia remains limited. Stress reduction through handling techniques has been shown to decrease induction drug requirements and optimize sedation times in cats [39]. Moreover, a preliminary study suggested that trazodone may reduce preoperative stress and propofol requirements in routine surgical procedures [40]. Although anxiety does not necessarily correlate directly with postoperative pain scores in dogs [41], its management remains clinically relevant for optimizing patient welfare and perioperative handling.

Recently, tasipimidine has emerged as a promising pharmacological alternative. Approved by the European Union in 2021, it is indicated for short-term relief of anxiety and fear associated with noise or separation from the owner. Furthermore, tasipimidine oral bioavailability allows effective oral administration by the owner [42].

Pharmacodynamically, tasipimidine acts as a highly selective α2A-adrenoceptor agonist, showing strong affinity and full agonist activity at this human receptor subtype, with markedly lower activity at α2B and α2C receptors. Its mechanism of action involves inhibition of adenylyl cyclase and neuronal calcium influx, together with increased potassium conductance, resulting in neuronal hyperpolarization and reduced excitability [43]. Currently, doses of 30 µg/kg are well tolerated and produce the desired anxiolytic effects in dogs [44].

Primary objectives of this study were to evaluate the effect of oral administration of 30 µg/kg tasipimidine on preoperative anxiety, handling quality and cardiovascular outcomes.

Secondary objectives focused on evaluating sedation level prior to induction, propofol requirements, intraoperative rescue analgesia (fentanyl) requirements, and recovery quality as reflected by the need for rescue sedation (dexmedetomidine).

We hypothesized that the inclusion of tasipimidine in the pre-anesthetic protocol would improve anxiety control and handling quality, reduce drug requirements (propofol and fentanyl), and preserve clinically acceptable hemodynamic function without requiring cardiovascular intervention in healthy bitches undergoing ovariectomy.

## 2. Materials and Methods

### 2.1. Animals

A total of 30 adult female dogs scheduled for elective ovariectomy at the Rof Codina Veterinary Teaching Hospital (RCVTH) between August 2025 and January 2026 were enrolled in this study. All dogs were client-owned and housed outdoors.

Animals were randomly allocated into three groups (*n* = 10). Treatment group A (GTa) received oral (PO) tasipimidine (30 µg/kg; Tessie, Orion Corporation, Espoo, Finland) followed by intramuscular (IM) dexmedetomidine (2.5 µg/kg; Dexdomitor, Ecuphar, Oostkamp, Belgium) combined with morphine (0.3 mg/kg, IM; Morphine, B. Braun, Barcelona, Spain). Treatment group B (GTb) received tasipimidine PO (30 µg/kg) followed by dexmedetomidine (5 µg/kg IM) combined with morphine (0.3 mg/kg, IM). The control group (GC) received an oral placebo followed by dexmedetomidine IM (5 µg/kg) combined with morphine (0.3 mg/kg, IM).

Tasipimidine or placebo was administered approximately 45 min before arrival at RCVTH, following a 6 h preoperative food fast and free access to water. Premedication was injected 15 min after arrival.

The study was conducted under a blind design; neither the clinician performing the evaluation nor the owner was aware of the treatment allocation. Group assignment was performed by an independent investigator who was not involved in data collection or outcome assessment. Written informed consent was obtained from all owners prior to inclusion, following a detailed explanation of the study protocol.

All procedures conducted on the study were approved by the Ethics Committee of the Galician Public Foundation Rof Codina (Reference number: AELU001/25/INVMED(02)/ANIMAL(05)/AGC/01).

A pre-appointment anamnesis was conducted with the animal owners to ensure that no concomitant diseases were present and that no ongoing treatments could interfere with the procedure. In addition, all animals had experienced at least one estrous cycle and were currently in the anestrus phase [45]. Furthermore, owners were asked to characterize the temperament of their animals, and fearfulness was recorded as a dichotomous variable (fearful vs. non-fearful) for subsequent analysis.

Animals were assigned an anesthetic risk category based on the American Society of Anesthesiologists (ASA) classification, and only those considered ASA I were eligible for inclusion. In addition, animals that did not allow completion of a full physical examination due to extreme aggressive behavior were excluded from the study. Furthermore, any animals that developed surgical or anesthetic complications during the procedures such as severe bradycardia (<30 bpm) and hypotension (systolic < 80 mm Hg, mean < 60 mm Hg, and diastolic < 40 mm Hg) [14,46,47] were consequently treated and excluded.

Two dogs were excluded from the study for not meeting the inclusion criteria. One dog was excluded after developing severe persistent bradycardia and hypotension during anesthesia; subsequent cardiological assessment revealed a previously undiagnosed third-degree atrioventricular block, confirming that the animal had a pre-existing cardiac condition incompatible with the inclusion criteria. The other was excluded due to extreme aggressive behavior that prevented completion of the general physical exam; this dog initially belonged to the control group. To maintain the predetermined sample size of 30 animals, these were replaced with two additional bitches.

### 2.2. Experimental Design

**Figure 1:**
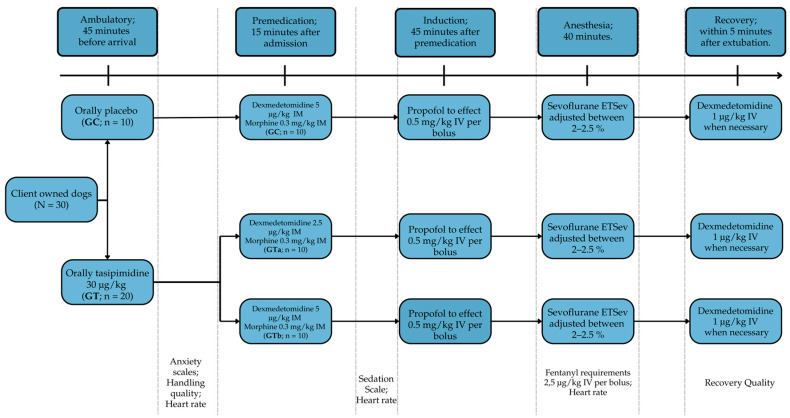
Flow chart depicting the experimental design and perioperative variables. Dogs were assigned to either GC (oral placebo + dexmedetomidine 5 µg/kg IM + morphine 0.3 mg/kg IM) or GT (oral tasipimidine 30 µg/kg). The GT was further divided into two subgroups: GTa (dexmedetomidine 2.5 µg/kg IM + morphine 0.3 mg/kg IM) and GTb (dexmedetomidine 5 µg/kg IM + morphine 0.3 mg/kg IM).

For the evaluation of variables prior to premedication, two groups were defined, GC and treatment group (TG) where GTa and GTb were included (Figure 1).

After admission dogs were transferred to a quiet, low-stimulus room. There, preoperative anxiety was evaluated using a reproduction of the Clinic Dog Stress Scale (CDSS) [34,48], which assesses 7 behavior variables (body posture, ears position, gaze, respiration, lip tension, activity and vocalization). Each outcome was scored from 0 to 4, except for lip tension, which was scored from 0 to 3. The total score was obtained by summing all parameter scores, resulting in a final scale ranging from 0 to 27, with higher scores indicating greater levels of stress. Additionally, a Visual Analog Scale (VAS) [49] was applied for subjective clinician assessment (0–10, with 10 representing very high anxiety), and a shortened and modified Reactivity Evaluation Form (REF) [41,50] scored dogs based on their behavior toward a directly approaching observer, ranging from 1 to 4 with higher scores reflecting stronger avoidance and refusal of contact (Table 1).

Following these assessments, a general physical exam (GPE) was conducted. Afterward, the same clinician responsible for the GPE was asked to rate the ease of performing the procedure using a reproduction of the Numerical Rating Scale (NRS), rating from 1 to 5, 1 = excellent (no resistance), 2 = good (minor resistance requiring standard restraint), 3 = fair (moderate resistance requiring increased restraint), 4 = poor (marked resistance requiring forceful restraint and/or additional personnel), and 5 = not possible (procedure could not be fully completed due to extreme resistance or stress-related behaviors) [38]. This allowed a subjective quantitative assessment of the animal’s compliance and the practical feasibility of the examination.

Then, animals received IM premedication approximately 15 min after admission, according to group allocation. At this point, the GT was subdivided based on the dexmedetomidine dose, as mentioned, into GTa (dexmedetomidine 2.5 µg/kg + morphine 0.3 mg/kg IM) and GTb (dexmedetomidine 5 µg/kg + morphine 0.3 mg/kg IM). Premedication consisted of dexmedetomidine and morphine administered in the same syringe.

Once light to moderate sedation had been achieved, a catheter was inserted into the cephalic vein to allow blood sampling and administration of intravenous (IV) Lactated Ringer’s solution (5 mL/kg/h; Lactato Ringer Vet, B. Braun). Blood sample was processed, analyzed and evaluated prior to induction. A single preoperative dose of meloxicam (0.2 mg/kg IV; Metacam, Boehringer Ingelheim, Barcelona, Spain) was given. Cefazolin (22 mg/kg IV; Cefazolin Normon, Normon Laboratories S.A., Madrid, Spain) was administered for antimicrobial prophylaxis prior to surgery and repeated after 90 min. Following preparation of the surgical field, the animals were moved to the operating room, where oxygen was delivered via face mask (3–4 L/min) for five minutes.

Pre-induction sedation was assessed 40–45 min after premedication, using the full version of the validated Sedation Scale (SS) [51,52], which evaluates spontaneous posture, palpebral reflex, eye position, jaw and tongue relaxation, response to noise, resistance to lateral recumbency, and general attitude. Scores ranged from 0 to 21, with higher values indicating deeper sedation. At this time, variables—including respiratory rate (RR, breaths/min), heart rate (HR, beats/min), non-invasive arterial pressure (NIAP) (SAP/DAP (MAP); mmHg), and temperature (T, °C)—were recorded.

Anesthesia was induced using propofol (0.5–3 mg/kg IV; Lipuro, B. Braun), administered as repeated boluses of 0.5 mg/kg every 15 s until conditions suitable for orotracheal intubation were achieved [53]. The cumulative dose required to obtain loss of palpebral and laryngeal reflexes, adequate jaw relaxation, and ventromedial rotation of the eye with mild mydriasis was recorded.

Then, tracheal intubation was performed in sternal recumbency. The tube was secured to the mandible with gauze.

Following intubation, animals were positioned in dorsal recumbency, and Intermittent Positive Pressure Ventilation (IPPV) was initiated using an anesthesia machine (Mindray WATO EX-35). The initial tidal volume was set at 10 mL/kg and subsequently adjusted to maintain normocapnia (EtCO_2_ = 35–45 mmHg). The respiratory rate was set at 12–14 breaths/min, with an inspiration-to-expiration ratio of 1:2, a positive end-expiratory pressure (PEEP) of 4 cm H_2_O, and an inspiratory pause of 25%. Anesthesia was maintained with sevoflurane in O_2_, with vaporizer adjusted and titrated according to clinical indicators including mandibular tone, ventromedial eye rotation, and absence of palpebral reflex [54].

Surgery consisted of a cranial midline infraumbilical laparotomy (3–6 cm incision, depending on animal size), performed by experienced soft tissue surgeons, with a surgical duration of approximately 25–30 min. Ovaries were exteriorized under gentle traction and resected using a harmonic scalpel (HARMONIC ACE^®^ +7 handpiece, Ethicon Endo-Surgery [Europe] GmbH, Norderstedt, Germany). After confirming hemostasis, a peritoneal cavity lavage with bupivacaine (2 mg/kg; Bupivacaina B. Braun) was performed. Then the incision was closed in three layers: muscle, subcutaneous tissue, and skin.

Variables—including EtCO_2_ (mmHg), end-tidal sevoflurane concentration (ET-Sev, %), RR (breaths/min), HR (beats/min), NIAP (SAP/DAP (MAP); mmHg), oxygen saturation (SpO2, %), and temperature (T, °C)—were recorded every 5 min using a multiparameter veterinary monitor (Mindray iPM12 Vet) configured in canine patient mode. HR was derived from the ECG signal, RR was measured by thoracic impedance respiration, NIAP was obtained using an appropriately sized cuff selected according to limb circumference and cuff markings, and temperature was measured using an esophageal probe connected to the temperature cable. Outcomes (HR and NIAP) related to six predefined intraoperative time points—T0 (baseline values recorded after induction and cardiorespiratory stabilization, prior to surgical stimulation), T1 (abdominal cavity opening), T2 (first ovarian pedicle traction), T3 (second ovarian pedicle traction), T4 (abdominal closure), and R (recovery)—were used for intergroup statistical comparisons.

Intraoperative nociceptive response was monitored every 5 min and defined as spontaneous breathing or ≥20% increases in SAP, DAP, MAP or HR from baseline (T0) sustained for more than 30 s [55]. Rescue analgesia consisted of fentanyl (2.5 µg/kg IV, Fentadon, Dechra, Barcelona, Spain).

Anesthetic recovery quality was assessed 5 min after extubation, using the Simple Descriptive Scale (SDS) to assess quality of recovery rating from 1 to 5, 5 being the poorest recovery quality [56,57]. The evaluation was based on direct clinical observation. Following this, rescue sedation treatment (dexmedetomidine; 1 µg/kg IV) was applied to those animals scoring ≥ 3 on SDS and were classified as agitated recoveries. Those scoring ≤ 2 on SDS were classified as good recoveries and did not receive rescue sedation.

The requirement for rescue sedation was prospectively recorded for each animal as a binary outcome (as mentioned) and subsequently analyzed to assess its association with treatment allocation.

After surgery, animals recovered under direct supervision and were transferred to the hospitalization ward once they were conscious, responsive, and considered clinically stable. Postoperative pain was routinely monitored by the hospitalization veterinarians responsible for patient care using the Glasgow Composite Measure Pain Scale–Short Form (CMPS-SF) [58], and analgesic treatment was adjusted according to clinical requirements until discharge. At discharge, all dogs received routine postoperative analgesia with a non-steroidal anti-inflammatory drug (NSAID), meloxicam 0.1 mg/kg PO, once a day for 7 days, and rescue analgesia (paracetamol 10 mg/kg PO; Apiretal, Ern S.A. Laboratories, Barcelona, Spain) was prescribed when considered necessary. In addition, postoperative follow-up was conducted by telephone to monitor recovery and provide guidance to the owners when required. Postoperative pain assessment formed part of the routine clinical management of all animals; however, pain scores and rescue analgesic requirements were not predefined study outcomes and were therefore not included in the statistical analysis.

### 2.3. Statistical Data Analyses

Sample size calculation was performed using G*Power software (version 3.1.9.7). An a priori power analysis was conducted using an F test for repeated-measures ANOVA (within–between interaction). The following parameters were applied: effect size (f) = 0.5, α = 0.05, statistical power (1 − β) = 0.8, three groups, six repeated measurements, and a correlation among HR repeated measures of 0.56 (data obtained from a previous study [40]). The calculated total sample size was 30 animals.

Parametric data are presented as mean ± SD and non-parametric data are presented as median and range. Statistical analyses were performed using SigmaPlot 12.5 (Systat Software Inc., Chicago, IL, USA). Normality of the data was assessed using the Shapiro–Wilk test, and homogeneity of variances was evaluated with Levene’s test.

For comparisons between two groups, Student’s *t*-test was used for normally distributed variables, and the Mann–Whitney U test was applied when normality and/or equality of variance assumptions were not met.

For comparisons among three groups, one-way ANOVA was performed for normally distributed variables, followed by the Holm–Sidak post hoc test, whereas for non-normally distributed variables, the Kruskal–Wallis test was used, followed by Dunn’s post hoc test.

Physiological variables measured at surgical time points (T0–T5) were analyzed using one-way repeated-measures ANOVA for normal variables, with the Holm–Sidak post hoc test. Non-normally distributed repeated measures were analyzed using the Friedman test, followed by Tukey post hoc test.

Categorical (dichotomous) variables were analyzed by calculating odds ratios (ORs) with 95% confidence intervals, and significance was assessed using the Chi-square or Fisher’s exact test, as appropriate.

A *p*-value < 0.05 was considered statistically significant.

## 3. Results

### 3.1. Animals Characteristics

The animals were females aged between 8 months and 6 years. Mean body weight was 20.06 ± 11.38 kg attending a normal distribution. BCS was 2.75 [2–4] in the GC, 2.5 [2–3] in the GTa, and 3 [2–3.5] in the GTb, with no statistically significant differences among groups (*p* = 0.26).

According to the owners’ assessment, 60% of the animals in the GC, 60% in the GTa, and 50% in the GTb were classified as fearful, with no significant differences between groups.

### 3.2. General Physical Exam

For the evaluation of variables prior to premedication, two groups were defined, the control group (GC) and treatment group (GT) where GTa and GTb were included.

**Figure 2:**
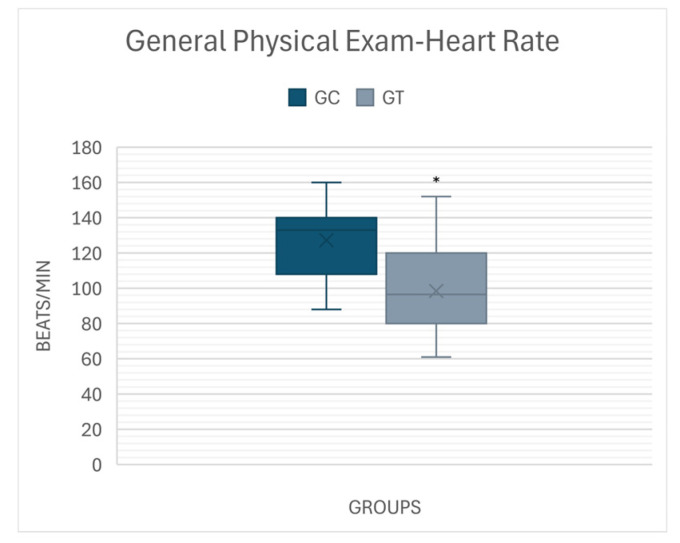
Heart rate (HR) measured at the Preoperative General Physical Examination. Box represents the interquartile range (IQR), horizontal line represents the median, X represents the mean, whiskers indicate the spread of the data (*n* = 10 for GC; *n* = 20 for GT). *p* < 0.05 * vs. GC.

General physical examination findings were within normal limits in all animals. Mean HR (Figure 2) was 127 ± 22 bpm in the GC and 84 ± 19 bpm in the GT, showing a statistically significant difference (*p* < 0.001; 95% confidence interval (CI) [18.60–59.69 bpm]).

### 3.3. Anxiety Variables and Handling Quality

Preoperative anxiety was assessed using three scales (Table 2).

**Figure 3:**
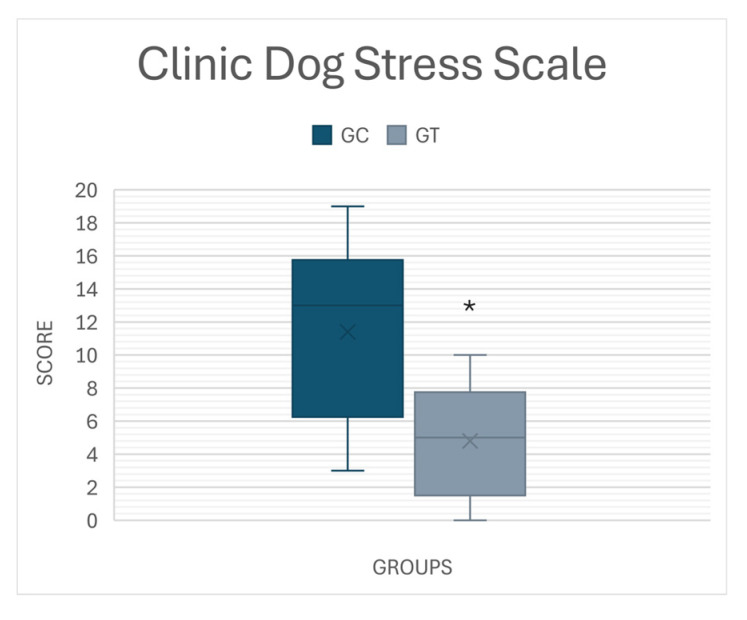
Anxiety scale. Clinic Dog Stress Scale (CDSS) score [0–27] comparison. Box represents the IQR, horizontal line represents the median, X represents the mean, whiskers indicate the spread of the data (*n* = 10 for GC; *n* = 20 for GT). *p* < 0.05 * vs. GC.

**Figure 4:**
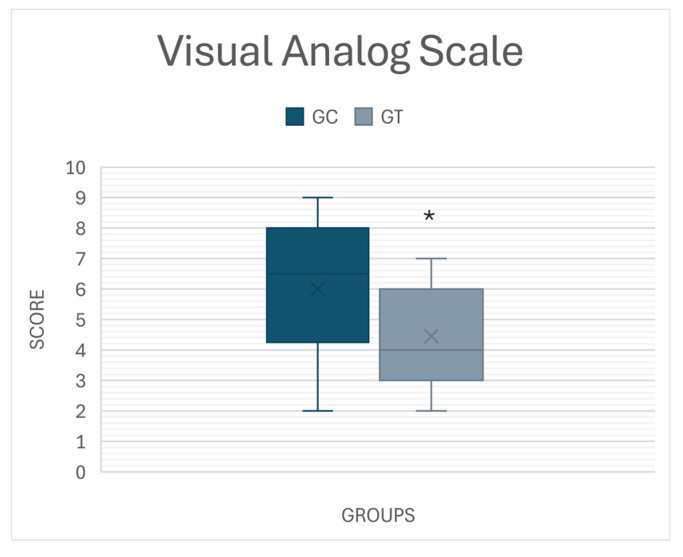
Anxiety scale. Visual Analog Scale (VAS) score [0–10] comparison. Box represents the IQR, horizontal line represents the median, X represents the mean, whiskers indicate the spread of the data (*n* = 10 for GC; *n* = 20 for GT). *p* < 0.05 * vs. GC.

The GT demonstrated significantly lower CDSS, VAS and REF scores compared with the GC (Figure 3 and Figure 4, Table 2).

**Figure 5:**
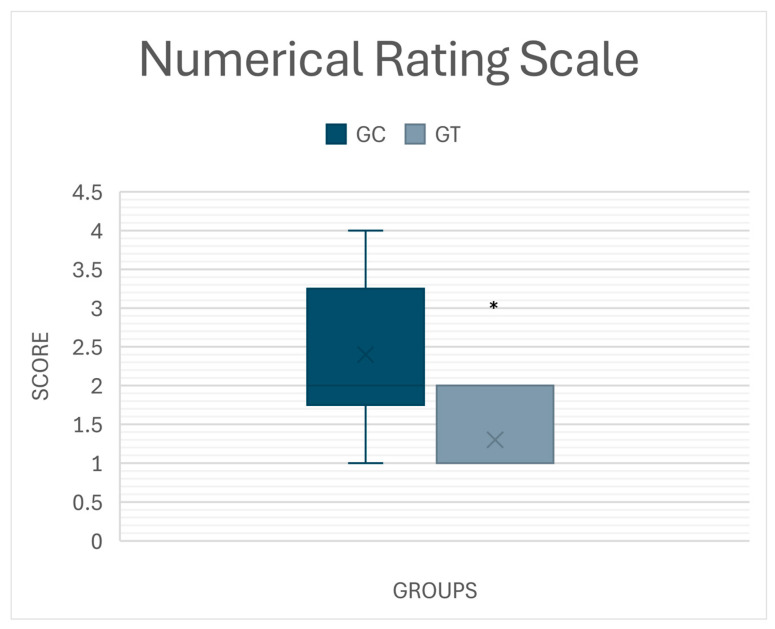
Numerical Rating Scale (NRS) score [1–5] comparison. Box represents IQR, horizontal line represents the median, X represents the mean, whiskers indicate the spread of the data (*n* = 10 for GC; *n* = 20 for GT). *p* < 0.05 * vs. GC.

The ability to perform the physical exam, assessed using the NRS (Figure 5), was significantly improved in the GT compared with the GC. The GC showed a median and range score of 2 [1–4], whereas the GT had a median and range score of 1 [1–2]. This difference was statistically significant (*p* = 0.003).

### 3.4. Premedication

After premedication, the GT was further subdivided according to the administered dexmedetomidine dose into GTa and GTb.

The interval between IM premedication and attainment of a sedation level adequate for venous catheter placement and initiation of IV fluid therapy was 14.9 ± 6.01 min in the GC, 11.0 ± 3.53 min in the GTa, and 14.3 ± 5.03 min in the GTb, with no statistically significant differences detected among groups (*p* = 0.18).

Emesis following IM premedication was recorded in 60% of animals in the GC and 60% in the GTb, whereas 30% of animals in the GTa exhibited vomiting after drug administration. The incidence of emesis did not differ significantly among groups (*p* = 0.33).

### 3.5. Induction Data and Induction Agent Requirements

**Figure 6:**
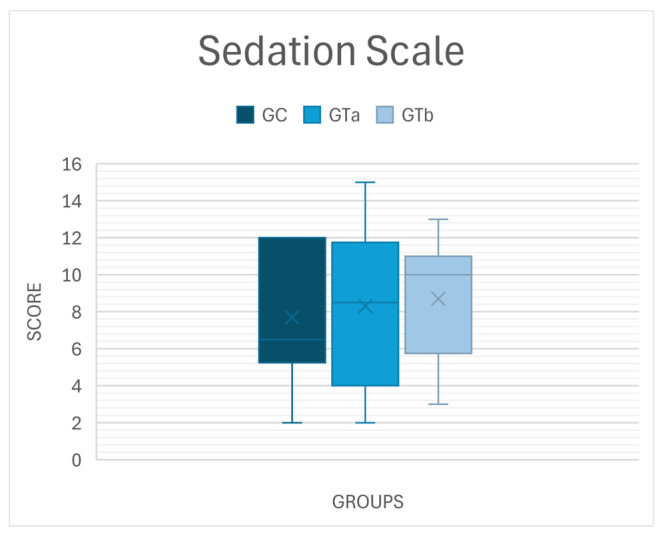
Sedation Scale (SS) score [0–20] comparison. Box represents IQR, horizontal line represents the median, X represents the mean, whiskers indicate the spread of the data (*n* = 10 for GC; *n* = 10 for GTa; *n* = 10 for GTb).

Before induction, SS scores (Figure 6) were 6.5 [2–12] in the GC, 8.5 [2–15] in the GTa, and 10 [3–13] in the GTb, without significant differences among groups (*p* = 0.970).

**Figure 7:**
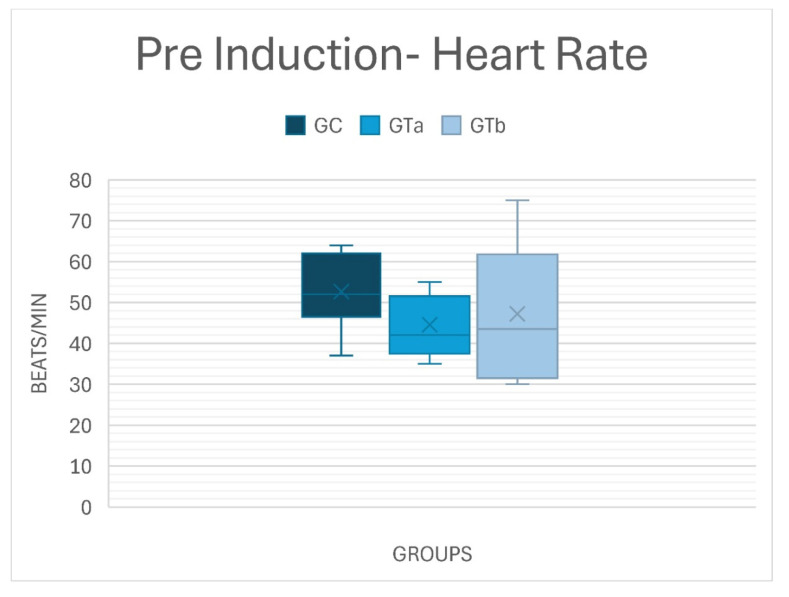
Heart rate (HR) prior to induction. Box represents IQR, horizontal line represents the median, X represents the mean, whiskers indicate the spread of the data (*n* = 10 for GC; *n* = 10 for GTa; *n* = 10 for GTb).

Regarding physiological variables recorded, HR (Figure 7) was 52 ± 9 bpm in the GC, 44 ± 8 bpm in the GTa, and 47 ± 17 bpm in the GTb (*p* = 0.21). RR was 16 ± 4 rpm in the GC, 13 ± 6 rpm in the GTa, and 16 ± 6 rpm in the GTb (*p* = 0.27). Rectal T values were 38.2 ± 0.5 °C, 38.2 ± 0.9 °C, and 38.3 ± 0.9 °C in the GC, GTa, and GTb, respectively (*p* = 0.57).

Prior to induction, SAP/DAP (MAP), mmHg values were 114 ± 14/81 ± 18 (91 ± 16) mmHg in the GC, 102 ± 19/71 ± 17 (79 ± 18) mmHg in the GTa, and 90 ± 24/58 ± 15 (67 ± 15) mmHg in the GTb. Statistically significant reductions in SAP, DAP, and MAP were observed in the GTb compared with the GC (*p* < 0.05 for all comparisons).

**Figure 8:**
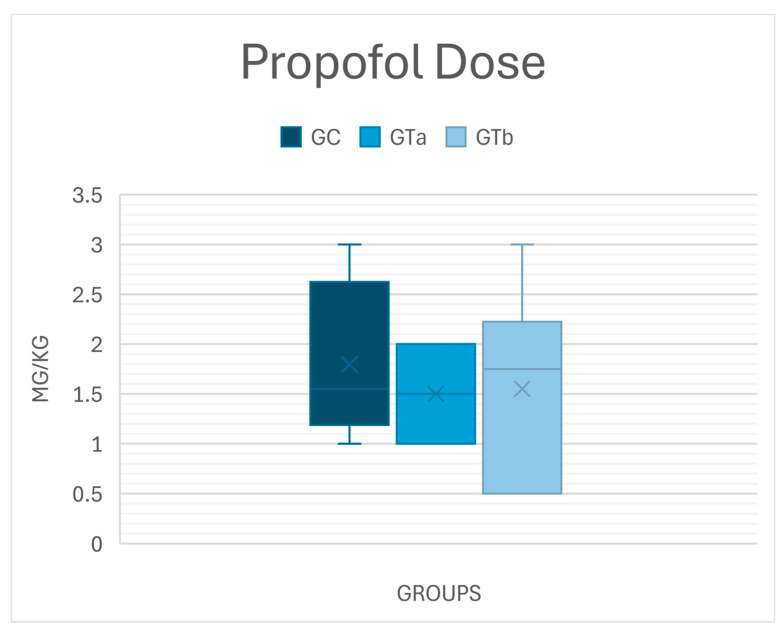
Propofol dose required (PDR) mg/kg [0.5–3] comparison. Box represents IQR, horizontal line represents the median, X represents the mean, whiskers indicate the spread of the data (*n* = 10 for GC; *n* = 10 for GTa; *n* = 10 for GTb).

The mean PDR (Figure 8) to achieve induction was 1.8 ± 0.8 mg/kg, 1.5 ± 0.5 mg/kg, and 1.5 ± 0.9 mg/kg for the GC, GTa, and GTb, respectively (*p* = 0.63).

### 3.6. Intraoperative Heart Rate and Non-Invasive Arterial Pressure Monitoring

Once the desired anesthetic plane for surgery was achieved, baseline intraoperative monitoring was recorded at T0. At this time point, HR was significantly lower in both the GTa 53 ± 16 bpm; *p* = 0.009 and GTb 56 ± 20 bpm; *p* = 0.021 compared with the basal (T0) HR of the GC 79 ± 15 bpm (Figure 7, Table 3).

**Figure 9:**
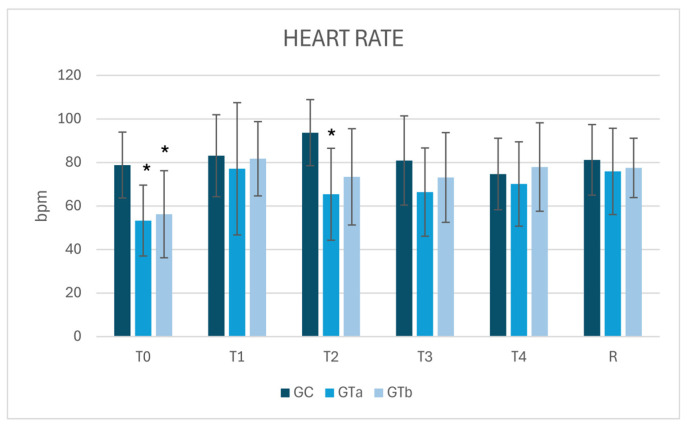
Heart rate (HR) measured at six predefined surgical time points: T0 (baseline), T1 (abdominal cavity opening), T2 (first ovarian pedicle traction), T3 (second ovarian pedicle traction), T4 (abdominal closure), and R (recovery). Values given as a mean ± SD. *p* < 0.05: * vs. GC within the same time point. (*n* = 10 for GC; *n* = 10 for GTa; *n* = 10 for GTb).

At T1 (Table 3), NIAP; SAP/DAP (MAP), mmHg differed significantly between the GC 95 ± 17/55 ± 16 (68 ± 14) and the GTb 110 ± 8/68 ± 7 (83 ± 5); *p* < 0.05. In addition, HR (Figure 9) in the GTb increased to 82 ± 17 bpm compared with its baseline, reaching statistical significance.

At T2 (Table 3), HR was significantly higher in the GC 94 ± 15 bpm than in the GTa 65 ± 21 bpm; *p* < 0.05. MAP also differed significantly between the GTa 97 ± 15 mmHg and the GTb 83 ± 9 mmHg; *p* < 0.05. Also, the increase in HR (Figure 9) observed in the GC relative to T0 at this time point was statistically significant (*p* < 0.05).

At T3 (Table 3), no remarkable changes in HR (Figure 9) or NIAP were observed between groups or compared with T0.

At T4 (Table 3), HR (Figure 9) increased significantly in the GTb 78 ± 20 bpm relative to its baseline (*p* < 0.05). Additionally, DAP differed significantly between the GC 58 ± 15 mmHg and the GTa 72 ± 11 mmHg; *p* < 0.05.

At R (Table 3) HR (Figure 9) increased significantly in GTa and GTb compared to its relative baseline (*p* < 0.05).

Additional intraoperative monitoring variables, including T, EtCO_2_ and ETSev are summarized in Supplementary Table S1. 

### 3.7. Intraoperative Rescue Analgesia

During surgery, the median and range of rescue analgesia boluses administered was 1 [0–3] in the GTa, 2 [0–3] in the GTb, and 1 [0–2] in the GC, with no statistically significant differences between groups (*p* = 0.104). Four animals in the GTa, one in the GTb, and two in the GC did not require any rescue boluses.

### 3.8. Recovery

**Figure 10:**
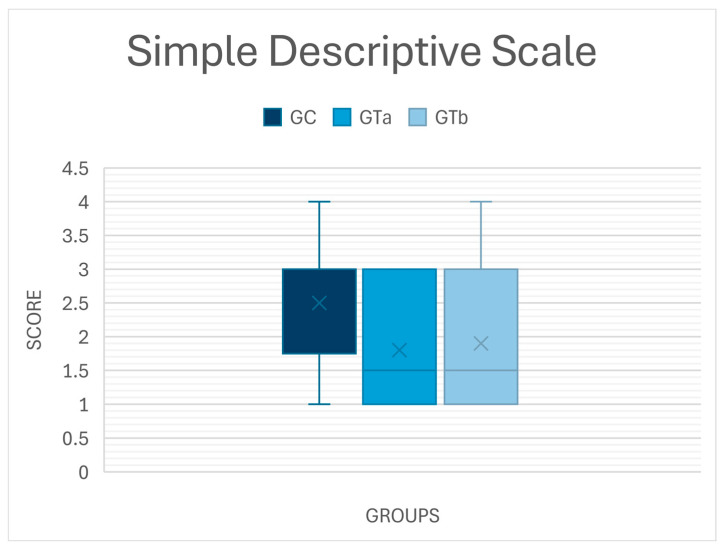
Simple Descriptive Scale (SS) score [1–5] comparison. Box represents IQR, horizontal line represents the median, X represents the mean, whiskers indicate the spread of the data (*n* = 10 for GC; *n* = 10 for GTa; *n* = 10 for GTb).

Upon recovery from anesthesia, SDS scores (Figure 10) were 3 [1–4] in the GC, 1.5 [1–3] in the GTa, and 1.5 [1–4] in the GTb. Without significant difference among groups (*p* = 0.242). Agitated recoveries requiring rescue sedation were observed in three animals in the GTa (30%), three animals in the GTb (30%), and six animals in the GC (60%). No statistically significant differences were observed among groups (*p* = 0.33).

## 4. Discussion

The present study suggested that oral administration of 30 µg/kg tasipimidine was associated with lower scores on the behavioral scales used and with improved handling quality in dogs undergoing elective ovariectomy. A decrease in HR prior to premedication, prior to induction and at the onset of baseline anesthesia was observed in tasipimidine-treated dogs. The addition of tasipimidine did not translate into higher pre-induction sedation levels, lower propofol requirements, or reduced intraoperative fentanyl requirements, but a reduction in dexmedetomidine requirements during recovery was observed, although statistical significance was not achieved. Overall, these findings support an improvement in observable anxiety-related behaviors and handling conditions. However, these behavioral improvements were not accompanied by measurable differences in sedation scores, anesthetic drug requirements, or intraoperative fentanyl rescue doses under the conditions of the present study. Heart rate values were lower in the tasipimidine-treated group compared with the control group during physical examination.

Tasipimidine administered at 30 µg/kg has been considered appropriate for inclusion in anesthetic protocols in healthy dogs, allowing titration of additional anesthetic agents to effect [59]. Its pharmacodynamic effects—including bradycardia, hypotension, anxiolysis, and mild sedation—are mediated primarily through activation of central α2A-adrenoceptors, resulting in a sympatholytic effect within the central nervous system [43].

Regarding anxiety-related behaviors and handling quality, oral tasipimidine was associated with significantly lower preoperative scores on the behavioral scales used in this study, which translated into improved handling by clinical staff during examination and preoperative preparation. The use of graded behavioral scales has been proposed as an appropriate method for quantifying behavioral alterations in dogs and provides a structured framework for assessing stress-related responses [34,38,51,60]. In this context, the observed reduction in anxiety-related behavioral scores is consistent with a potential anxiolytic effect of tasipimidine. However, because the CDSS, VAS, and REF rely on subjective behavioral assessment and have not been formally validated for the specific quantification of perioperative anxiety, these findings should be interpreted with caution. Therefore, the present results support an improvement in observable stress-related behaviors and handling conditions, which, considering the anxiolytic profile of tasipimidine, may suggest a potential anxiolytic effect. Further studies incorporating validated behavioral instruments and objective physiological markers of stress are warranted to better characterize the anxiolytic effects of tasipimidine in the perioperative setting [34].

The evaluation of oral dexmedetomidine gel—a selective α2-adrenoceptor agonist with anxiolytic properties [61]—have demonstrated its ability to reduce anxiety and increase tolerance to external stimuli. Furthermore, the association between reduced anxiety and decreased responsiveness to stressors [62] supports the interpretation that the stress-reducing effects observed in the present investigation reflect anxiolysis. Similarly, oral dexmedetomidine gel has been reported to provide significant clinical benefits. Specifically, treatment facilitated physical examination at both 125 µg/kg and 250 µg/kg doses [38].  These findings are aligned with the present results, which demonstrated improved manageability following tasipimidine administration. Although mild bradycardia and pale mucous membranes were observed at the higher treatment dose, according to the authors no clinical safety concerns were reported [38].

In the present study, a statistically significant reduction in HR was observed during the GPE in treated animals compared with controls. Reductions in MAP (−18 ± 3 mmHg) and HR (−55 ± 10 bpm) following tasipimidine 30 µg/kg administration were reported and it was suggested that calmer animals may exhibit more pronounced bradycardia [44]; these findings aligned with those of our study.

Because the tasipimidine-treated animals were subsequently assigned to two dexmedetomidine dosing regimens, post-premedication outcomes should be interpreted in the context of the complete anesthetic protocol administered to each group. This is particularly relevant for variables such as HR and NIAP, as these variables may be affected by the different dexmedetomidine doses administered in each group. Lower HR values were recorded in the tasipimidine-treated groups throughout the procedure. Moreover, a statistically significant decrease in NIAP was recorded in GTb compared to GC prior to induction. Although significant differences were detected in HR and NIAP at specific time points, the clinical relevance of these changes remains uncertain. The observed differences were relatively modest and were not accompanied by other objective indicators of cardiovascular function, such as cardiac output, tissue perfusion, or invasive arterial pressure measurements. Consequently, while the results confirm that tasipimidine administration was associated with some measurable physiological effects, the extent to which these statistically significant differences translate into clinically meaningful cardiovascular consequences cannot be determined from the present data.

Regarding the potential pharmacological mechanisms underlying the bradycardia observed in the present study, several factors associated with α2-adrenergic agonist administration should be considered. The lower HR observed in tasipimidine-treated groups is compatible with the known pharmacological effects of α2-adrenergic agonists, which act on the vasomotor centers of the rostral ventrolateral medulla, producing vasodilation and bradycardia, likely mediated by increased parasympathetic activity [63,64,65,66]. After premedication, bradycardia was observed in all groups, particularly prior to induction, whereas no significant increase in MAP was detected, including in groups receiving the higher dexmedetomidine dose (GC and GTb). Although the absence of a concomitant increase in NIAP may not strongly support a predominantly baroreflex-mediated mechanism [64], the present data do not allow the underlying mechanism of the observed bradycardia to be determined. Both central sympatholytic effects and peripheral cardiovascular effects associated with α2-adrenergic agonists may have contributed to the cardiovascular responses observed [67,68,69]. Experimental studies in dogs have shown that α2-agonist administration may markedly decrease cardiac output despite relatively preserved arterial blood pressure, likely due to compensatory increases in systemic vascular resistance. Furthermore, coadministration of vatinoxan with medetomidine has been associated with improved hemodynamic performance and less pronounced reductions in cardiac output, supporting the importance of peripheral vascular effects in α2-agonist-induced cardiovascular changes [70,71]. Therefore, because cardiac output, systemic vascular resistance, and tissue perfusion were not directly assessed, the present cardiovascular findings should be interpreted cautiously and should not be considered a comprehensive assessment of cardiovascular function.

As with HR and NIAP, the interpretation of sedation, anesthetic requirement, and recovery quality outcomes should consider the potential influence of the different dexmedetomidine doses administered in addition to tasipimidine.

The addition of oral tasipimidine to a 5 µg/kg IM dexmedetomidine–0.3 mg/kg IM morphine premedication protocol did not result in a higher degree of sedation compared with oral placebo followed by the same premedication protocol. However, animals receiving tasipimidine followed by a lower dexmedetomidine dose (GTa; 2.5 µg/kg IM dexmedetomidine-0.3 mg/kg IM morphine) achieved a comparable level of sedation and required similar propofol doses than the control group. Both tasipimidine and dexmedetomidine produce anxiolysis and sedation, respectively [43]. However, contrary to expectations, the addition of tasipimidine to a dexmedetomidine–morphine premedication protocol did not result in deeper sedation immediately prior to anesthetic induction (between 40 and 45 min after premedication).

Regarding propofol dose requirements, previous findings have indicated that the addition of tasipimidine, compared with a control group receiving only placebo treatments (oral placebo followed by IV saline), resulted in a significant reduction in propofol requirements [72]. In contrast, in the present study the control group did not receive the placebo alone but was premedicated IM as mentioned. Under these study conditions, although the tasipimidine-treated groups required on average 0.3 mg/kg less propofol than the placebo group, this difference did not reach statistical significance.

Based on the expected anxiolytic and sympatholytic effects of adding tasipimidine to a dexmedetomidine–morphine premedication protocol, we hypothesized that improved perioperative anxiolysis could potentially influence intraoperative fentanyl rescue requirements. The human literature has suggested a possible association between stress-related mechanisms and pain perception, including hypotheses proposing stress as a contributing factor in recurrent pain in pediatric populations [73]. In addition, a clinical study in humans evaluating buccal dexmedetomidine gel reported sedative and hemodynamic effects and postoperative analgesic outcomes; however, analgesia was assessed in the postoperative period [74], whereas the present study focused specifically on intraoperative nociceptive responses. In veterinary medicine, a randomized clinical trial in dogs undergoing laparoscopic ovariectomy compared opioid-free and opioid-sparing protocols and implemented rescue dexmedetomidine (0.5 µg/kg IV) in response to predefined cardiovascular thresholds (>20% increase from baseline), illustrating an alternative approach to intraoperative nociceptive modulation [75]. Reductions in fentanyl requirements would be clinically relevant, given the potential adverse effects of fentanyl, including bradycardia, hypotension, delayed anesthetic recovery, and dysphoric reactions [76,77]. Nevertheless, in the present study, no statistically significant differences in intraoperative fentanyl requirements were observed between groups.

The onset of action of tasipimidine has been described at approximately 15–20 min after administration, with peak effects around 1 h and a duration of action extending up to 4 h [43]. In the present study, approximately 2 h and 45 min elapsed between oral administration of the drug and anesthetic recovery. Therefore, it can be reasonably assumed that tasipimidine was still exerting anxiolytic effects during the recovery period. Regarding recovery quality, six out of 20 animals in the tasipimidine-treated groups (GTa and GTb) required rescue sedation with IV dexmedetomidine (1 µg/kg) due to agitated emergence. In comparison, six out of 10 animals in the control group required the same intervention. These findings suggest that, although rescue sedation was still necessary in a proportion of treated animals, the incidence of agitated recovery appeared proportionally lower in the tasipimidine-treated groups than in control group.

As discussed, the reduction in anxiety-related behavioral scores observed after tasipimidine administration was not accompanied by significant differences in sedation scores, propofol requirements, intraoperative fentanyl administration or recovery quality. This observation may indicate that improvement of preoperative anxiety-related behaviors does not necessarily translate into measurable anesthetic- or analgesic-sparing effects within the context of a multimodal anesthetic protocol. From a clinical perspective, facilitating patient handling and reducing observable signs of stress may constitute relevant outcomes on their own, even when no clear reduction in anesthetic drug requirements is detected. Nevertheless, the active premedication administered to all groups and the fact that the study was powered on HR rather than these secondary outcomes should be considered when interpreting these results.

The limitations of this study should be acknowledged. First, as tasipimidine was administered at the animals’ homes prior to hospital admission, correct administration and full compliance could not be completely verified. Second, the pharmacokinetic and pharmacodynamic profiles of tasipimidine were not directly assessed; therefore, the timing of administration was based on previously published data [42,43]. Third, interactions between tasipimidine and other drugs could not be completely assessed, as all groups included dexmedetomidine in the premedication protocol. Fourth, the propofol induction protocol, based on repeated boluses administered every 15 s, may have influenced the total induction dose required, as slower administration rates could potentially allow better assessment of the peak clinical effect. Fifth, the specific relationship between fentanyl administration and the observed cardiovascular changes was not formally analyzed. Sixth, the study was statistically powered based on HR as the primary physiological variable; therefore, secondary outcomes may have been underpowered to detect smaller between-group differences, and the absence of statistically significant differences in some variables should be interpreted cautiously. Finally, cardiovascular effects were only partially evaluated, since variables assessing invasive arterial pressure, tissue perfusion and cardiac output were not included and could have provided a more accurate representation of cardiovascular function. Consequently, further research is warranted to better characterize the clinical use of tasipimidine and other anxiolytic agents in dogs undergoing surgery, particularly regarding optimal dosing strategies, safety, postoperative analgesia, and their overall impact on perioperative welfare.

## 5. Conclusions

Under the conditions of the present study, preoperative oral administration of tasipimidine was associated with reduced anxiety-related behavioral scores and improved handling quality in healthy dogs undergoing elective ovariectomy. Although tasipimidine administration was accompanied by lower heart rate values, the clinical relevance of these cardiovascular changes remains uncertain, particularly because cardiovascular function was not comprehensively assessed. Furthermore, the behavioral improvements observed were not associated with clear differences in sedation, anesthetic requirements, or recovery quality. Further investigations are warranted to better define the clinical relevance of tasipimidine as part of perioperative anesthetic protocols and to determine whether its behavioral effects confer additional benefits beyond improved patient manageability.

## Figures and Tables

**Table 1:** Reactivity Evaluation Form. Scored dogs based on their behavior toward a directly approaching observer.

Reactivity Evaluation Form (Adapted)	
1	The dog actively seeks contact and remains positioned continuously in front of the veterinarian.
2	The dog remains in close proximity to the veterinarian, occasionally retreating or backing away slightly.
3	The dog tends to remain distant from the veterinarian, although occasional attempts to seek contact are observed.
4	The dog remains distant from the veterinarian and does not seek any contact.

Reactivity Evaluation Form (REF) adapted and shortened [41,50].

**Table 2:** Anxiety scales’ values.

Anxiety Scales	GC	GT
CDSS	13 [3–19]	* 5 [0–10]
VAS	6.5 [2–9]	* 4 [2–7]
REF	2 [1–4]	* 1 [1–3]

Variables evaluated in 30 bitches undergoing ovariectomy. CDSS (Clinic Dog Stress Scale), VAS (Visual Analog Scale), REF (Reactivity Evaluation Form), (*n* = 10 for GC; *n* = 20 for GT). Values given as median and [range]. *p* < 0.05 * vs. GC.

**Table 3:** Intraoperative HR and NIAP variables.

		GC	GTa	GTb
HR (bpm)	T0	79 ± 15	* 53 ± 16	* 56 ± 20
	T1	83 ± 19	77 ± 30	^a^ 82 ± 17
	T2	^a^ 94 ± 15	* 65 ± 21	73 ± 22
	T3	81 ± 20	66 ± 20	73 ± 21
	T4	75 ± 16	^c^ 70 ± 19	^a^ 78 ± 20
	R	80 ± 16	^a^ 76 ± 20	^a^ 77 ± 14
SAP (mmHg)	T0	105 ± 13	107 ± 8	110 ± 15
	T1	95 ± 17	107 ± 14	*110 ± 8
	T2	^b^ 115 ± 15	123 ± 17	112 ± 11
	T3	108 ± 18	115 ± 20	114 ± 14
	T4	93 ± 20	^c^ 106 ± 14	106 ± 13
	R	102 ± 14	106 ± 16	103 ± 11
DAP (mmHg)	T0	75 ± 10	71 ± 12	70 ± 11
	T1	^a^ 55 ± 16	67 ± 13	* 68 ± 7
	T2	^b^ 80 ± 13	^b^ 84 ± 15	** 71 ± 9
	T3	^b^ 72 ± 18	80 ± 18	70 ± 12
	T4	^a^ 58 ± 15	^c,^* 72 ± 11	61 ± 10
	R	69 ± 18	75 ± 17	62 ± 12
MAP (mmHg)	T0	82 ± 10	81 ± 12	82 ± 12
	T1	68 ± 14	80 ± 14	* 83 ± 5
	T2	^b^ 90 ± 11	^b^ 97 ± 15	** 83 ± 9
	T3	^b^ 85 ± 17	90 ± 19	87 ± 10
	T4	71 ± 19	81 ± 13	78 ± 7
	R	80 ± 18	85 ± 16	75 ± 11

Variables evaluated in 30 bitches undergoing ovariectomy, values given as mean ± SD. *p* < 0.05: ^a^ vs. T0, ^b^ vs. T1, ^c^ vs. T2 within same group, * vs. GC same time point, ** vs. GTa same time point.

## Data Availability

The original contributions presented in this study are included in the article. Further inquiries can be directed to the corresponding authors.

## Supplementary Materials

The following supporting information can be downloaded at https://www.mdpi.com/article/10.3390/vetsci13070618/s1, Table S1: Intraoperative values: end-tidal CO_2_, temperature and end-tidal Sevoflurane.

## Abbreviations

The following abbreviations are used in this manuscript:

ASA	American Society of Anesthesiologists
CI	Confidence Interval
CMPS-SF	Glasgow Composite Measure Pain Scale–Short Form
DAP	Diastolic Arterial Pressure
EtCO_2_	End Tidal CO_2_
ETSev	End Tidal Sevoflurane concentration
GC	Control Group
GPE	General Physical Exam
GT	Treatment Group
GTa	Treatment Group A
GTb	Treatment Group B
HR	Heart Rate
IM	Intramuscular
IPPV	Intermittent Positive Pressure Ventilation
IQR	Interquartile Range
IV	Intravenous
MAP	Mean Arterial Pressure
NIAP	Non-Invasive Arterial Pressure
NRS	Numerical Rating Scale
NSAID	Non-Steroidal Anti-Inflammatory Drug
OR	Odds Ratio
PDR	Propofol Dose Required
PO	Oral
RCVTH	Rof Codina Veterinary Teaching Hospital
REF	Reactivity Evaluation Form
RR	Respiratory Rate
SAP	Systolic Arterial Pressure
SDS	Simple Descriptive Scale
SpO2	Oxygen Saturation
SS	Sedation Scale
T	Temperature
VAS	Visual Analog Scale

## References

[1] Hernander L. (2009). Factors Influencing Dogs’ Stress Level in the Waiting Room at a Veterinary Clinic.

[2] Wolfle T.L. (2000). Understanding the Role of Stress in Animal Welfare: Practical Considerations. The Biology of Animal Stress: Basic Principles and Implications for Animal Welfare.

[3] Dinwoodie I.R., Dwyer B., Zottola V., Gleason D., Dodman N.H. (2019). Demographics and Comorbidity of Behavior Problems in Dogs. J. Vet. Behav..

[4] Kooriyama T., Ogata N. (2021). Salivary Stress Markers in Dogs: Potential Markers of Acute Stress. Res. Vet. Sci..

[5] Tiira K., Sulkama S., Lohi H. (2016). Prevalence, Comorbidity, and Behavioral Variation in Canine Anxiety. J. Vet. Behav. Clin. Appl. Res..

[6] Crociolli G.C., Cassu R.N., Barbero R.C., Rocha T.L.A., Gomes D.R., Nicácio G.M. (2015). Gabapentin as an Adjuvant for Postoperative Pain Management in Dogs Undergoing Mastectomy. J. Vet. Med. Sci..

[7] Aghighi S.A., Tipold A., Piechotta M., Lewczuk P., Kästner S.B.R. (2012). Assessment of the Effects of Adjunctive Gabapentin on Postoperative Pain after Intervertebral Disc Surgery in Dogs. Vet. Anaesth. Analg..

[8] Oyenekan I.O., Osunlakin J.O., Adeoyo K., Makinde A.O., Kehinde O.F., Ajadi R.A. (2025). Evaluation of Preoperative Gabapentin on Cardiopulmonary Parameters and Postoperative Pain in Dogs Undergoing Elective Ovariohysterectomy. Folia Vet..

[9] Johnson B.A., Aarnes T.K., Wanstrath A.W., Ricco Pereira C.H., Bednarski R.M., Lerche P., McLoughlin M.A. (2019). Effect of Oral Administration of Gabapentin on the Minimum Alveolar Concentration of Isoflurane in Dogs. Am. J. Vet. Res..

[10] Aiello J., Carson B., Aarnes T., Wanstrath A., McLoughlin M. (2025). Effect of Oral Premedication with Gabapentin and Trazodone Combination on the MAC of Isoflurane in Dogs. Am. J. Vet. Res..

[11] Brosnan R.J., Pypendop B.H., Cenani A. (2024). Effects of Trazodone and Dexmedetomidine on Fentanyl-Mediated Reduction of Isoflurane Minimum Alveolar Concentration in Cats. Vet. Anaesth. Analg..

[12] Murphy L.A., Barletta M., Graham L.F., Reichl L.J., Duxbury M.M., Quandt J.E. (2017). Effects of Acepromazine and Trazodone on Anesthetic Induction Dose of Propofol and Cardiovascular Variables in Dogs Undergoing General Anesthesia for Orthopedic Surgery. J. Am. Vet. Med. Assoc..

[13] Hoffman E.A., Aarnes T.K., Pereira C.H.R., Lerche P., Bednarski R.M., McLoughlin M.A. (2018). Effect of Oral Trazodone on the Minimum Alveolar Concentration of Isoflurane in Dogs. Vet. Anaesth. Analg..

[14] Grubb T., Sager J., Gaynor J.S., Montgomery E., Parker J.A., Shafford H., Tearney C. (2020). Anesthesia and Monitoring Guidelines for Dogs and Cats. J. Am. Anim. Hosp. Assoc..

[15] Friedrich S., Reis S., Meybohm P., Kranke P. (2022). Preoperative Anxiety. Curr. Opin. Anaesthesiol..

[16] Wang R., Huang X., Wang Y., Akbari M. (2022). Non-Pharmacologic Approaches in Preoperative Anxiety, a Comprehensive Review. Front. Public Health.

[17] Kisielewska W., Kościółek M., Kowalczyk W., Mitura B., Mitura L., Rogula S., Leszczyński P.K., Antosik K., Mitura K. (2025). Decreasing Preoperative Anxiety in Patients with Newly Available Multimodal Approaches-A Narrative Review. J. Clin. Med..

[18] Liu W., Xu R., Jia J., Shen Y., Li W., Bo L. (2022). Research Progress on Risk Factors of Preoperative Anxiety in Children: A Scoping Review. Int. J. Environ. Res. Public Health.

[19] Shebl M.A., Toraih E., Shebl M., Tolba A.M., Ahmed P., Banga H.S., Orz M., Tammam M., Saadalla K., Elsayed M. (2025). Preoperative Anxiety and Its Impact on Surgical Outcomes: A Systematic Review and Meta-Analysis. J. Clin. Transl. Sci..

[20] Dantas L.M.S., Ogata N. (2024). Veterinary Psychopharmacology. Vet. Clin. N. Am. Small Anim. Pract..

[21] Dreschel N.A. (2010). The Effects of Fear and Anxiety on Health and Lifespan in Pet Dogs. Appl. Anim. Behav. Sci..

[22] Jeyaretnam J., Jones H., Phillips M. (2000). Disease and Injury among Veterinarians. Aust. Vet. J..

[23] Nordgren L.D., Gerberich S.G., Alexander B.H., Church T.R., Bender J.B., Ryan A.D. (2014). Evaluation of Factors Associated with Work-Related Injuries to Veterinary Technicians Certified in Minnesota. J. Am. Vet. Med. Assoc..

[24] Fu V.X., Oomens P., Sneiders D., van den Berg S.A.A., Feelders R.A., Wijnhoven B.P.L., Jeekel J. (2019). The Effect of Perioperative Music on the Stress Response to Surgery: A Meta-Analysis. J. Surg. Res..

[25] Li H., Cheng M., Jiang S. (2025). The Impact of Music Therapy on Preoperative Anxiety in Pediatric Patients Undergoing Anesthesia: A Systematic Review and Meta-Analysis. J. Perianesthesia Nurs..

[26] Suleiman-Martos N., García-Lara R.A., Membrive-Jiménez M.J., Pradas-Hernández L., Romero-Béjar J.L., Dominguez-Vías G., Gómez-Urquiza J.L. (2022). Effect of a Game-Based Intervention on Preoperative Pain and Anxiety in Children: A Systematic Review and Meta-Analysis. J. Clin. Nurs..

[27] Stamenkovic D.M., Rancic N.K., Latas M.B., Neskovic V., Rondovic G.M., Wu J.D., Cattano D. (2018). Preoperative Anxiety and Implications on Postoperative Recovery: What Can We Do to Change Our History. Minerva Anestesiol..

[28] Torres-González M.I., Manzano-Moreno F.J., Vallecillo-Capilla M.F., Olmedo-Gaya M.V. (2020). Preoperative Oral Pregabalin for Anxiety Control: A Systematic Review. Clin. Oral Investig..

[29] Shimony N., Amit U., Minz B., Grossman R., Dany M.A., Gonen L., Kandov K., Ram Z., Weinbroum A.A. (2016). Perioperative Pregabalin for Reducing Pain, Analgesic Consumption, and Anxiety and Enhancing Sleep Quality in Elective Neurosurgical Patients: A Prospective, Randomized, Double-Blind, and Controlled Clinical Study. J. Neurosurg..

[30] Maitra S., Baidya D.K., Bhattacharjee S., Som A. (2017). Perioperative Gabapentin and Pregabalin in Cardiac Surgery: A Systematic Review and Meta-analysis. Braz. J. Anesthesiol..

[31] Hammerle M., Horst C., Levine E., Overall K., Radosta L., Rafter-Ritchie M., Yin S. (2015). 2015 AAHA Canine and Feline Behavior Management Guidelines. J. Am. Anim. Hosp. Assoc..

[32] Lloyd J.K.F. (2017). Minimising Stress for Patients in the Veterinary Hospital: Why It Is Important and What Can Be Done about It. Vet. Sci..

[33] Moffat K. (2008). Addressing Canine and Feline Aggression in the Veterinary Clinic. Vet. Clin. N. Am. Small Anim. Pract..

[34] King T., Flint H.E., Hunt A.B.G., Werzowa W.T., Logan D.W. (2022). Effect of Music on Stress Parameters in Dogs during a Mock Veterinary Visit. Animals.

[35] Wakana K., Kimura Y., Nitta Y., Fujisawa T. (2022). The Effect of Music on Preoperative Anxiety in an Operating Room: A Single-Blind Randomized Controlled Trial. Anesth. Prog..

[36] Kim S.A., Borchardt M.R., Lee K., Stelow E.A., Bain M.J. (2022). Effects of Trazodone on Behavioral and Physiological Signs of Stress in Dogs during Veterinary Visits: A Randomized Double-Blind Placebo-Controlled Crossover Clinical Trial. J. Am. Vet. Med. Assoc..

[37] Erickson A., Harbin K., MacPherson J., Rundle K., Overall K.L. (2021). A Review of Pre-Appointment Medications to Reduce Fear and Anxiety in Dogs and Cats at Veterinary Visits. Can. Vet. J..

[38] Korpivaara M., Huhtinen M., Aspegrén J., Overall K. (2021). Dexmedetomidine Oromucosal Gel Reduces Fear and Anxiety in Dogs during Veterinary Visits: A Randomised, Double-blind, Placebo-controlled Clinical Pilot Study. Vet. Rec..

[39] Argüelles J., Echaniz M., Bowen J., Fatjó J. (2021). The Impact of a Stress-Reducing Protocol on the Quality of Pre-Anaesthesia in Cats. Vet. Rec..

[40] Cambeiro-Camarero N., Fernández-Martín S., González-Cantalapiedra A. (2025). A Preliminary Study: Evaluation of Oral Trazodone as a Strategy to Reduce Anesthetic Requirements in Bitches Undergoing Ovariectomy. Animals.

[41] Ellwood B., Murison P.J. (2022). Investigating the Effect of Anxiety on Pain Scores in Dogs. Vet. Anaesth. Analg..

[42] Tessie 0.3 Mg/Ml—Oral Solution|UPD. https://medicines.health.europa.eu/veterinary/es/600000004664.

[43] Lehtimäki J., Jalava N., Unkila K., Aspegren J., Haapalinna A., Pesonen U. (2022). Tasipimidine—The Pharmacological Profile of a Novel Orally Active Selective A2A-Adrenoceptor Agonist. Eur. J. Pharmacol..

[44] Lindstedt J.M., Kirjavainen M.H., Levijoki J., Häggström J., Korpivaara M. (2025). Concurrent Use of Tasipimidine Oral Solution and Clomipramine in Dogs. J. Vet. Pharmacol. Ther..

[45] Romagnoli S., Krekeler N., de Cramer K., Kutzler M., McCarthy R., Schaefer-Somi S. (2024). WSAVA Guidelines for the Control of Reproduction in Dogs and Cats. J. Small Anim. Pract..

[46] Tranquilli W.J., Thurmon J.C., Grimm K.A., Lumb W.V. (2007). Lumb & Jones’ Veterinary Anesthesia and Analgesia.

[47] Duke-Novakovski T., de Vries M., Seymour C. (2016). BSAVA Manual of Canine and Feline Anaesthesia and Analgesia.

[48] Overall K. (2013). Manual of Clinical Behavioral Medicine for Dogs and Cats (VetBooks.Ir).

[49] Srithunyarat T., Hagman R., Höglund O.V., Stridsberg M., Hanson J., Lagerstedt A.S., Pettersson A. (2018). Catestatin, Vasostatin, Cortisol, and Visual Analog Scale Scoring for Stress Assessment in Healthy Dogs. Res. Vet. Sci..

[50] Palestrini C., Minero M., Cannas S., Berteselli G., Scaglia E., Barbieri S., Cavallone E., Puricelli M., Servida F., Dall’Ara P. (2010). Efficacy of a Diet Containing Caseinate Hydrolysate on Signs of Stress in Dogs. J. Vet. Behav. Clin. Appl. Res..

[51] Grint N.J., Burford J., Dugdale A.H.A. (2009). Does Pethidine Affect the Cardiovascular and Sedative Effects of Dexmedetomidine in Dogs?. J. Small Anim. Pract..

[52] Wagner M.C., Hecker K.G., Pang D.S.J. (2017). Sedation Levels in Dogs: A Validation Study. BMC Vet. Res..

[53] Raszplewicz J., Macfarlane P., West E. (2013). Comparison of Sedation Scores and Propofol Induction Doses in Dogs after Intramuscular Premedication with Butorphanol and Either Dexmedetomidine or Medetomidine. Vet. Anaesth. Analg..

[54] Ribeiro L.M., Ferreira D.A., Brás S., Gonzalo-Orden J.M., Antunes L.M. (2012). Correlation between Clinical Signs of Depth of Anaesthesia and Cerebral State Index Responses in Dogs with Different Target-Controlled Infusions of Propofol. Vet. Anaesth. Analg..

[55] Bradbrook C.A., Clark L., Dugdale A.H.A., Burford J., Mosing M. (2013). Measurement of Respiratory System Compliance and Respiratory System Resistance in Healthy Dogs Undergoing General Anaesthesia for Elective Orthopaedic Procedures. Vet. Anaesth. Analg..

[56] Lozano A.J., Brodbelt D.C., Borer K.E., Armitage-Chan E., Clarke K., Alibhai H.I.K. (2009). A Comparison of the Duration and Quality of Recovery from Isoflurane, Sevoflurane and Desflurane Anaesthesia in Dogs Undergoing Magnetic Resonance Imaging. Vet. Anaesth. Analg..

[57] Jones H., Robson K., Maddox T., Alderson B. (2024). Incidence of and Risk Factors for Poor Recovery Quality in Dogs Recovering from General Anaesthesia—A Prospective Case Control Study. Vet. Anaesth. Analg..

[58] Reid J., Nolan A.M., Hughes J.M.L., Lascelles D., Pawson P., Scott E.M. (2007). Development of the Short-Form Glasgow Composite Measure Pain Scale (CMPS-SF) and Derivation of an Analgesic Intervention Score. Anim. Welf..

[59] Kästner S.B., Amon T., Tünsmeyer J., Noll M., Söbbeler F.J., Laakso S., Saloranta L., Huhtinen M. (2024). Effects of Tasipimidine Premedication with and without Methadone and Dexmedetomidine on Cardiovascular Variables during Propofol-Isoflurane Anaesthesia in Beagle Dogs. Vet. Anaesth. Analg..

[60] Gruen M.E., Sherman B.L. (2008). Use of Trazodone as an Adjunctive Agent in the Treatment of Canine Anxiety Disorders: 56 Cases (1995-2007). J. Am. Vet. Med. Assoc..

[61] Messenger K.M., Hopfensperger M., Knych H.K., Papich M.G. (2016). Pharmacokinetics of Detomidine Following Intravenous or Oral-Transmucosal Administration and Sedative Effects of the Oral-Transmucosal Treatment in Dogs. Am. J. Vet. Res..

[62] Gruen M., Case B.C., Robertson J.B., Campbell S., Korpivaara M.E. (2020). Evaluation of Repeated Dosing of a Dexmedetomidine Oromucosal Gel for Treatment of Noise Aversion in Dogs over a Series of Noise Events. Vet. Rec..

[63] Nguyen V., Tiemann D., Park E., Salehi A. (2017). Alpha-2 Agonists. Anesthesiol. Clin..

[64] Bispo G.A., Cabral de Oliveira T., Moretti M.F., Soares M.F., Linhares L.C.M., de Souza N.S.N., da Costa A.K.S., Taffarel M.O., Ferreira W.L., dos Santo P.S.P. (2025). Dexmedetomidine in Healthy Dogs: Impact on Electrocardiographic Parameters. Vet. Med. Int..

[65] de Sant’Ana Alves L., Arcoverde K.N., de Oliveira C.V.A., Cavalcante J.M., Araújo-Silva G., de Paula V.V. (2024). Pharmacokinetics and Pharmacodynamics of Intravenous Dexmedetomidine (2 Μg∙kg^−1^) in Dogs. Res. Vet. Sci..

[66] Weerink M.A.S., Struys M.M.R.F., Hannivoort L.N., Barends C.R.M., Absalom A.R., Colin P. (2017). Clinical Pharmacokinetics and Pharmacodynamics of Dexmedetomidine. Clin. Pharmacokinet..

[67] Lemke K.A. (2004). Perioperative Use of Selective Alpha-2 Agonists and Antagonists in Small Animals. Can. Vet. J..

[68] Norman K., Nappe T.M. (2023). Alpha Receptor Agonist Toxicity. StatPearls.

[69] Congdon J.M., Marquez M., Niyom S., Boscan P. (2011). Evaluation of the Sedative and Cardiovascular Effects of Intramuscular Administration of Dexmedetomidine with and without Concurrent Atropine Administration in Dogs. J. Am. Vet. Med. Assoc..

[70] Turunen H., Raekallio M.R., Honkavaara J.M., Restitutti F., Kallio-Kujala I.J., Adam M., Nevanperä K., Scheinin M., Männikkö S.K., Hautajärvi H.J. (2019). Cardiovascular and Sedation Reversal Effects of Intramuscular Administration of Atipamezole in Dogs Treated with Medetomidine Hydrochloride with or without the Peripheral A2-Adrenoceptor Antagonist Vatinoxan Hydrochloride. Am. J. Vet. Res..

[71] Joerger F.B., Wieser M.L., Steblaj B., Niemann L., Turunen H., Kutter A.P. (2023). Evaluation of Cardiovascular Effects of Intramuscular Medetomidine and a Medetomidine–Vatinoxan Combination in Beagle Dogs: A Randomized Blinded Crossover Laboratory Study. Vet. Anaesth. Analg..

[72] Kästner S.B., Amon T., Tünsmeyer J., Noll M., Söbbeler F.J., Laakso S., Saloranta L., Huhtinen M. (2024). Anaesthetic-Sparing Effect of the Anxiolytic Drug Tasipimidine in Beagle Dogs. Vet. Anaesth. Analg..

[73] Alfven G., Grillner S., Andersson E. (2019). Review of Childhood Pain Highlights the Role of Negative Stress. Acta Paediatr..

[74] Mohamed S.A.B., Abdel-Ghaffar H.S., Hassan N.A.A., El Sherif F.A., Shouman S.A., Omran M.M., Hassan S.B., Allam A.A.A.E.M., Sayed D.G. (2021). Pharmacokinetics and Pharmacodynamics of 3 Doses of Oral-Mucosal Dexmedetomidine Gel for Sedative Premedication in Women Undergoing Modified Radical Mastectomy for Breast Cancer. Anesth. Analg..

[75] Lazzarini E., Gioeni D., Del Prete G., Sala G., Baio M., Carotenuto A.M. (2024). A Comparative Analysis of Opioid-Free and Opioid-Sparing Anaesthesia Techniques for Laparoscopic Ovariectomy in Healthy Dogs. Vet. Anaesth. Analg..

[76] Joshi G.P. (2023). Rational Multimodal Analgesia for Perioperative Pain Management. Curr. Pain Headache Rep..

[77] Epstein M., Rodan I., Griffenhagen G., Kadrlik J., Petty M., Robertson S., Simpson W. (2015). Pain Management Guidelines for Dogs and Cats. J. Am. Anim. Hosp. Assoc..

