# Supplementary material for: Preoperative Oral Tasipimidine in Dogs Undergoing Elective Ovariectomy: Anxiolysis, Handling Quality and Cardiovascular Effects
Source: Vet Sci. 2026 Jun 26;13(7):618. doi: 10.3390/vetsci13070618 (PMC13417972; doi:10.3390/vetsci13070618)
Supplement: Supplementary file 1 [file vetsci-13-00618-s001.zip › vetsci-4348837-supplementary.pdf]

Table S1: Intraoperative values: end-tidal CO_2, temperature and end-tidal Sevoflurane.

		GC	GTa	GTb
$EtCO_2$ (mmHg)	T0	43 ± 6	46 ± 4	41 ± 6
	T1	41 ± 2	45 ± 3	41 ± 5
	T2	$^{a, b}$ 38 ± 4	43 ± 4	41 ± 3
	T3	40 ± 4	43 ± 4	42 ± 4
	T4	46 ± 18	46 ± 3	^{c} 46 ± 3
	R	^{c} 41 ± 3	^{c} 48 ± 3	44 ± 4
T (ºC)	T0	37.5 ± 0.6	37.5 ± 0.8	37.5 ± 0.6
	T1	37.1 ± 0.8	37.4 ± 1.0	37.4 ± 0.9
	T2	36.9 ± 1.0	37.4 ± 1.1	37.2 ± 1.0
	T3	^{a} 36.7 ± 1.0	37.3 ± 1.0	37.1 ± 1.3
	T4	^{a} 36.6 ± 1.0	37.3 ± 1.0	37.2 ± 1.1
	R	$^{a, b}$ 36.6 ± 1.0	37.4 ± 1.1	36.4 ± 2.8
ETSev (%)	T0	2.49 ± 0.13	*1.87 ± 0.66	2.28 ± 0.62
	T1	2.58 ± 0.16	*2.11 ± 0.33	*2.23 ± 0.32
	T2	2.61 ± 0.315	*2.22 ± 0.3	2.42 ± 0.22
	T3	2.54 ± 0.4	2.36 ± 0.19	2.48 ± 0.27
	T4	2.44 ± 0.33	2.28 ± 0.19	2.43 ± 0.29
	R	--	--	--

Outcomes evaluated in 30 bitches undergoing ovariectomy, values given as mean ± SD. $p < 0.05$: ^{a} vs. T0, ^{b} vs. T1, ^{c} vs. T2 within same group, * vs. GC same time point, ** vs. GTa same time point.
